# Association of Programmed Cell Death Ligand 1 Expression Status With Receipt of Immune Checkpoint Inhibitors in Patients With Advanced Non–Small Cell Lung Cancer

**DOI:** 10.1001/jamanetworkopen.2020.7205

**Published:** 2020-06-08

**Authors:** Michael S. Leapman, Carolyn J. Presley, Weiwei Zhu, Pamela R. Soulos, Kerin B. Adelson, Rebecca A. Miksad, Daniel J. Boffa, Cary P. Gross

**Affiliations:** 1 Department of Urology, Yale University School of Medicine, New Haven, Connecticut; 2 Cancer Outcomes, Public Policy and Effectiveness Research Center, Yale University School of Medicine, New Haven, Connecticut; 3 The Ohio State University Comprehensive Cancer Center, Columbus

## Abstract

**Importance:**

Initial approval for immune checkpoint inhibitors (ICIs) for treatment of advanced non–small cell lung cancer (NSCLC) was limited to patients with high levels of programmed cell death ligand 1 (PD-L1) expression. However, in the period after approval, it is not known how new evidence supporting efficacy of these treatments in patients with low or negative PD-L1 expression was incorporated into real-world practice.

**Objective:**

To evaluate the association between PD-L1 testing and first-line ICI use.

**Design, Setting, and Participants:**

This retrospective cohort study (January 1, 2011, to December 31, 2018) used a deidentified nationwide electronic health record–derived database reflecting real-world care at more than 280 US community and academic cancer clinics (approximately 800 sites of care). Patients included those with advanced NSCLC without other identifiable variations diagnosed in the period after the US Food and Drug Administration’s initial first-line approval of ICIs for patients with high PD-L1 expression (≥50%).

**Exposure:**

First-line ICI treatment.

**Main Outcomes and Measures:**

Patterns of PD-L1 testing and first-line ICI treatment among all patients and patients stratified by tumor histologic type (squamous vs nonsquamous).

**Results:**

A total of 45 631 patients (mean [SD] age, 68.4 [9.6] years; 21 614 [47.4%] female) with advanced NSCLC were included in the study. PD-L1 testing increased from 468 (7.2%) in 2015 to 4202 (73.2%) in 2018. Within a subset of 7785 patients receiving first-line treatment in the period after first-line approval of pembrolizumab, those who received PD-L1 testing had a greater odds of receiving an ICI (odds ratio, 2.11; 95% CI, 1.89-2.36). Among patients with high PD-L1 expression (≥50%), 1541 (83.5%) received first-line ICI treatment; 776 patients (40.3%) with low PD-L1 expression (1%-49%) and 348 (32.3%) with negative PD-L1 expression (0%) also received ICIs. In addition, 755 untested patients (32.8%) were treated with a first-line ICI. The proportion of patients who received ICIs without PD-L1 testing increased during the study period (59 [17%] in quarter 4 of 2016 to 141 [53.8%] in quarter 4 of 2018).

**Conclusions and Relevance:**

In this study, use of first-line ICI treatment increased among patients with advanced NSCLC with negative, low, or untested PD-L1 expression status in 2016 through 2018. These findings suggest that national practice was rapidly responsive to new clinical evidence rather than adhering to regulatory guidance in place at the time.

## Key Points

**Question:**

How was programmed cell death ligand 1 (PD-L1) testing used to select immune checkpoint inhibitor treatment for patients with advanced non–small cell lung cancer (NSCLC)?

**Findings:**

In this cohort study of 45 631 patients with advanced NSCLC, substantial increases occurred in the use of first-line immune checkpoint inhibitor treatment among patients with low or negative PD-L1 expression and those without documented PD-L1 testing. These increases were consistent with the dissemination of new evidence supporting benefit in these groups.

**Meaning:**

The findings suggest that use and interpretation of the PD-L1 biomarker to guide first-line treatment for advanced NSCLC was rapidly responsive to new clinical evidence.

## Teaser

This cohort study evaluates the association between programmed cell death ligand 1 testing and first-line immune checkpoint inhibitor use in patients with advanced non–small cell lung cancer.

## Introduction

The US Food and Drug Administration (FDA) first approved pembrolizumab, an immune checkpoint inhibitor (ICI) that targets programmed cell death 1 (PD-1), as first-line treatment for advanced non–small cell lung cancer (NSCLC) on October 24, 2016.^1,2,3^ This landmark approval was based on the results of a phase 3 randomized clinical trial including untreated patients with advanced NSCLC whose tumors highly expressed programmed cell death ligand 1 (PD-L1) (expression ≥50%).^4^ On the basis of that study,^4^ the FDA’s initial indication for first-line ICI treatment was restricted to patients with PD-L1 expression of 50% or greater. In the period after approval, evidence of clinical efficacy at lower PD-L1 expression levels emerged in a series of studies (eTable 1 in the Supplement),^4,5,6,7,8,9,10,11^ ultimately leading to broadened approval without restriction to PD-L1 status. There was rapid adoption of pembrolizumab in the period after first-line approval, primarily among patients with high PD-L1 expression.^12^ However, it remains unknown whether national practice subsequently reflected emerging clinical evidence (ie, first-line ICI treatment regardless of PD-L1 expression) vs the existing guidance that remained in place (ie, ICI treatment only in subsets of patients with PD-L1 expression).

There are important reasons to examine whether clinical practice was associated with recommended PD-L1 testing of patients with advanced NSCLC in the period before the FDA’s broadened approval. First, the extent to which ICI treatment was administered to patients with low or negative PD-L1 expression is unknown but may have been motivated by new evidence as it became available.^5,7^ Second, there was a high level of enthusiasm for ICIs that may have led to increasing use regardless of PD-L1 status.^13,14^ Third, because ICIs have lower treatment efficacy in patients with negative or low PD-L1 expression, it is not known whether use of these agents varies across strata of PD-L1 expression. Whether ICIs were favored as monotherapy or in conjunction with other conventional therapies, such as cytotoxic chemotherapy, for patients with low or negative PD-L1 expression also remains unexplored but may have implications for therapeutic efficacy.

An expanding number of agents are now approved in conjunction with companion diagnostic tests that seek to tailor treatments to a specific patient population.^15,16^ However, little is known about how diagnostic tests have been used to direct novel therapies, particularly those accompanied by considerable enthusiasm. Evaluating the responsiveness of national practice to evolving evidence can, therefore, reveal insights about the contributions of new data, regulatory guidance, patient factors, and preferences. Within this context, we aimed to understand how PD-L1 testing has been integrated into the care of patients with advanced NSCLC in the period after pivotal approvals for ICIs. We sought to evaluate the association between PD-L1 testing and first-line treatment delivered in the real-world setting. We hypothesized that national practice patterns would reflect evolving clinical evidence, preceding changes to regulatory guidance and leading to increased ICI use in patients with advanced NSCLC.

## Methods

### Study Design

We performed a retrospective cohort study of patients diagnosed with advanced NSCLC from January 1, 2011, to December 31, 2018, in the Flatiron Health Database. Deidentified data from the Flatiron Health Database were stored in a Health Insurance Portability and Accountability Act–compliant and secure format. Institutional review board approval of the study protocol, with waiver of informed consent, was obtained before study conduct from the Copernicus Institutional Review Board, and the Yale University Institutional Review Board granted an exemption. This study followed the Strengthening the Reporting of Observational Studies in Epidemiology (STROBE) reporting guideline.^17^

The primary objective was to examine the proportion of patients receiving any form of first-line ICI treatment associated with their PD-L1 expression testing status. The secondary objectives were (1) to identify trends in and factors associated with PD-L1 testing associated with the first-line approval of pembrolizumab and (2) to evaluate how the results of PD-L1 testing were incorporated into the first-line treatment of advanced NSCLC.

### Data Source

We used the Flatiron Health Database, a deidentified, longitudinal, demographically and geographically diverse database derived from electronic health record (EHR) information. The database draws from more than 280 US cancer clinics (approximately 800 sites of care), representing more than 2.2 million patients with cancer available for analysis. Patients with advanced NSCLC in the Flatiron Health Database are demographically similar to those in the US population as represented in the Surveillance, Epidemiology, and End Results database.^14^ Deidentified patient-level data for the study were centrally integrated, harmonized, and aggregated by Flatiron Health in a manner agnostic to the source EHR. The patient-level data in the EHRs include structured data in addition to unstructured data collected via technology-enabled medical record abstraction from physician’s notes and other unstructured documents (eg, biomarker reports).^18,19^ Data provided for this study were deidentified, and provisions were in place to prevent reidentification to protect patients’ confidentiality. Rigorous quality control for structured and unstructured data was conducted for this data set.^20,21^

### Cohort Selection

We constructed 2 analytic samples to evaluate: trends in PD-L1 testing and changes in ICI treatment. The samples were derived from a cohort of 53 096 patients diagnosed with advanced NSCLC during 2011 to 2018 (eFigure 1 in the Supplement). To ensure that patients were actively receiving care within the Flatiron Health Database, we excluded those without evidence of an interaction with the health care system within 90 days of advanced diagnosis or survival less than 30 days after advanced diagnosis date. The resulting sample of 45 631 patients was used to assess the adoption of PD-L1 testing over time. To examine changes that occurred after first-line approval, we restricted this sample to patients diagnosed from October 1, 2016, to December 31, 2018. We excluded patients with known driver alterations (*ALK* [OMIM 105590], *EGFR* [OMIM 131550], or *ROS1* [OMIM 165020]), which would dictate alternative forms of treatment, patients who received some form of treatment before PD-L1 testing (if tested), and patients who did not receive NSCLC-directed treatment or unspecified clinical trial drugs, leaving a sample of 7785 patients.

### Study Variables

Patient demographic, clinical, and socioeconomic characteristics included age, sex, race/ethnicity, insurance status, tumor histologic type, clinical stage as assessed by the treating physician or Flatiron Health Database abstractor (American Joint Committee on Cancer), Eastern Cooperative Oncology Group (ECOG) performance status, comorbidity, smoking status, year of diagnosis, and receipt of molecular testing. For patients with missing insurance status, we imputed Medicare insurance for those 65 years or older given historically high rates of enrollment.^22^ *International Classification of Diseases, Ninth Revision (ICD-9)* and *International Statistical Classification of Diseases and Related Health Problems, Tenth Revision (ICD-10)* diagnosis codes were used to calculate modified Elixhauser comorbidity scores.^23^ Patients with ECOG scores of 3 or higher were regarded as having poor performance status, and those with Elixhauser scores of 3 or higher were considered to have high comorbidity.

The data set includes detailed information about the PD-L1 testing status and results and several other molecular markers with therapeutic implications for advanced NSCLC, including *ALK*, *EGFR*, and *ROS1*. Among patients with documented PD-L1 expression expressed continuously (ie, 0%-100%), we categorized results as negative (0%), low (1%-49%), or high (≥50%). Patients with documented PD-L1 testing were regarded as tested, whereas those without documentation of PD-L1 testing within the data set were regarded as untested. We compiled lines of therapy administered based on antineoplastic drugs with therapeutic indication for advanced NSCLC, categorized as follows: PD-1 axis ICI (pembrolizumab, atezolizumab, durvalumab, or nivolumab), cytotoxic chemotherapy, or other (including nonapproved anti cytotoxic T-lymphocyte–associated protein 4 checkpoint inhibitors). Lastly, we classified ICI use as monotherapy or in combination with other regimens.

### Statistical Analysis

We compiled clinical and demographic characteristics and compared the distributions between PD-L1 tested and untested patients using descriptive statistics. We characterized first-line treatment by PD-L1 testing status and compared distributions of treatment by PD-L1 testing status and expression level. We evaluated time trends in the use of PD-L1 testing in the entire period and centered around the fourth quarter of 2016 when regulatory approval for pembrolizumab in the first line for advanced NSCLC was granted. We used multivariable logistic regression to examine the associations between clinical and demographic characteristics and use of PD-L1 testing.

**Figure 1.**
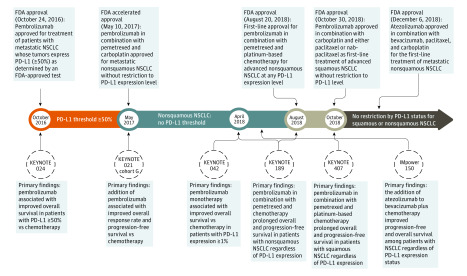
Timeline of Pivotal Trials and First-Line Approvals for Immune Checkpoint Inhibitor Therapy in Advanced Non–Small Cell Lung Cancer (NSCLC) FDA indicates US Food and Drug Administration; PD-L1, programmed cell death ligand 1.

We characterized the association of first-line treatment with regulatory guidance by assessing the proportion of patients who received ICIs in the first line across strata of PD-L1 testing status. Approved indications for first-line ICI treatment changed during the study period by tumor histologic type (squamous vs nonsquamous) and manner of treatment (ie, monotherapy or in combination with other agents) (Figure 1). Therefore, we evaluated trends in the use of ICI therapy by PD-L1 testing status across disease histologic type and whether ICIs were given as monotherapy. We used logistic regression to examine the independent association between PD-L1 testing results and use of first-line ICI, adjusted for clinical and demographic factors that may modify treatment decisions. We performed a sensitivity analysis among patients who may be preferentially treated with ICI because of poor performance status (ECOG score ≥3) or high comorbidity (≥3 conditions). We assessed statistical significance using 2-sided tests at an α level of .05. All analyses were performed using SAS software, version 9.4 (SAS Institute Inc).

## Results

### Adoption of PD-L1 Testing

A total of 45 631 patients (mean [SD] age, 68.4 [9.6] years; 21 614 [47.4%] female) with advanced NSCLC were included in the study (Table 1). The proportion of patients with PD-L1 testing increased rapidly after regulatory approval for pembrolizumab in October 2016 (eFigure 2 in the Supplement). In 2015, 468 patients (7.2%) underwent PD-L1 testing within 30 days of diagnosis, increasing to 4202 (73.2%) in 2018. Overall, 5482 eligible patients (70.4%) received PD-L1 testing, and 4848 (88.4%) had test results with a continuous (0%-100%) proportion of PD-L1 staining available. Female sex (odds ratio [OR], 1.22; 95% CI, 1.10-1.36) and nonsquamous histologic type (OR, 1.68; 95% CI, 1.34-2.10) were associated with greater odds of PD-L1 testing (eTable 2 in the Supplement).

**Table 1.** Characteristics of the Study Cohorts of Patients With Advanced Non–Small Cell Lung Cancer^a^

Characteristic	Cohort 1: patients diagnosed January 1, 2011 to December 31, 2018 (n = 45 631)	Cohort 2: patients diagnosed in the era of first-line immune checkpoint inhibitor approval (October 1, 2016 to December 31, 2018) (n = 7785)
Age, median (IQR), y	70 (62-77)	70 (62-77)
Sex		
Male	24 017 (52.6)	4351 (55.9)
Female	21 614 (47.4)	3434 (44.1)
Race/ethnicity		
Non-Hispanic white	31 160 (68.3)	5267 (67.7)
Non-Hispanic black	3849 (8.4)	689 (8.9)
Hispanic/Latino	1511 (3.3)	252 (3.2)
Asian	1077 (2.4)	127 (1.6)
Other	3394 (7.4)	605 (7.8)
Missing	4640 (10.2)	845 (10.9)
Insurance		
Medicare and other	19 616 (43.0)	3881 (49.9)
Medicare only	3702 (8.1)	565 (7.3)
Medicare, unknown if other or alone	8544 (18.7)	1063 (13.7)
Commercial health plan	5064 (11.1)	953 (12.2)
Medicaid	484 (1.1)	77 (1.0)
Other	2241 (4.9)	450 (5.8)
Uninsured or unknown	5980 (13.1)	796 (10.2)
Histologic type		
NSCLC histologic type NOS	2468 (5.4)	379 (4.9)
Non–squamous cell carcinoma	31 511 (69.1)	5112 (65.7)
Squamous cell carcinoma	11 652 (25.5)	2294 (29.5)
Stage at initial diagnosis		
I	3929 (8.6)	605 (7.8)
II	2376 (5.2)	415 (5.3)
III	9243 (20.3)	1664 (21.4)
IV	27 974 (61.3)	4516 (58.0)
Not reported or occult	2109 (4.6)	585 (7.5)
ECOG score		
0	9564 (21.0)	2059 (26.5)
1	11 890 (26.1)	2670 (34.3)
2	4798 (10.5)	1008 (13.0)
3-4	1461 (3.2)	230 (3.0)
Missing	17 918 (39.3)	1818 (23.4)
Elixhauser comorbidities		
0	40 534 (88.8)	6687 (85.9)
1-2	3977 (8.7)	826 (10.6)
≥3	1120 (2.5)	272 (3.5)
Smoking status		
Yes	39 648 (86.9)	7196 (92.4)
No	5426 (11.9)	578 (7.4)
Unknown	557 (1.2)	11 (0.1)
Molecular testing		
* ALK*	27 591 (60.5)	5540 (71.2)
* EGFR*	29 329 (64.3)	5637 (72.4)
* KRAS*	13 685 (30.0)	3329 (42.8)
* ROS1*	16 222 (35.6)	4863 (62.5)

Abbreviations: ECOG, Eastern Cooperative Oncology Group; IQR, interquartile range; NOS, not otherwise specified; NSCLC, non–small cell lung cancer.

^a^ Data are presented as number (percentage) of patients unless otherwise indicated.

### Association of PD-L1 Expression With First-Line Treatment

**Figure 2.**
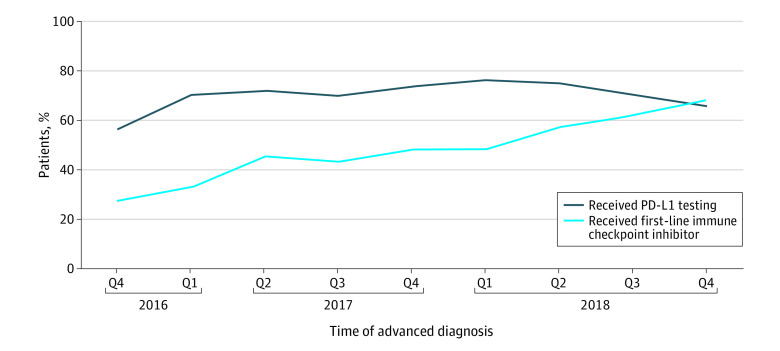
Trends in Programmed Cell Death Ligand 1 (PD-L1) Testing and Receipt of First-Line Immune Checkpoint Inhibitor Treatment Among Patients With Advanced Non–Small Cell Lung Cancer Q indicates quarter.

**Figure 3.**
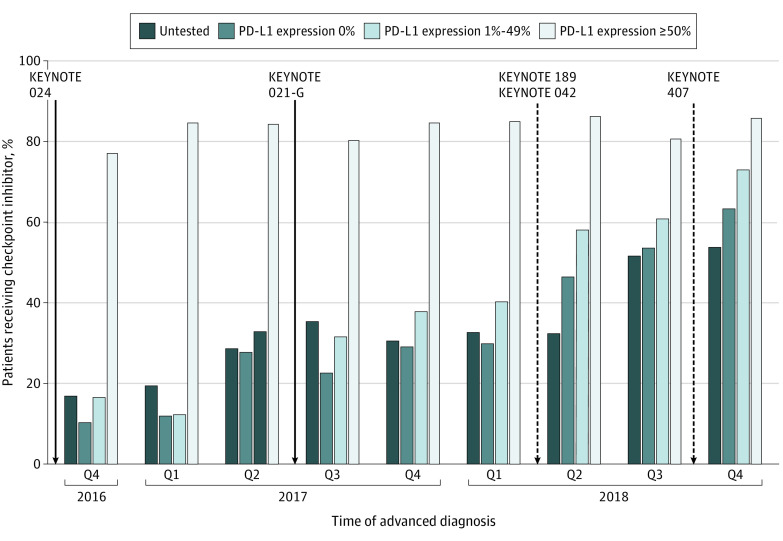
Association Between Publication of Clinical Trials That Addressed Efficacy of First-Line Pembrolizumab and Quarterly Percentage of Patients Receiving Immune Checkpoint Inhibitor Therapy by Programmed Cell Death Ligand 1 (PD-L1) Expression Status Restriction of pembrolizumab to patients with PD-L1 expression of 50% or greater was based on evidence from KEYNOTE 024. Subsequent evidence supported efficacy of pembrolizumab at lower PD-L1 expression (KEYNOTE 042) and regardless of PD-L1 expression (KEYNOTE 021 cohort G, 189, and 407). Q indicates quarter.

The overall proportion of patients treated with a first-line ICI increased during the study period (Figure 2). Patients who received PD-L1 testing were more frequently treated with an ICI (2975 [54.3%]) compared with untested patients (755 [32.8%]). Among patients with PD-L1 expression of 50% or greater, 1541 (83.5%) received ICI treatment; however, ICI use was also common in untested patients and those with expression levels below the regulatory threshold at the time (Figure 3). Among patients with negative PD-L1 expression (0% staining), 348 (32.3%) received an ICI in the first line, as did 755 (32.8%) of untested patients (Table 2). Compared with untested patients, those who received PD-L1 testing had greater odds of receiving ICI treatment in the first line (OR, 2.11; 95% CI, 1.89-2.36). Sensitivity analyses revealed that performance status and comorbidity were not associated with differential use of ICI treatment.

**Table 2.** First-Line Therapy Received by PD-L1 Expression Category Among 7785 Patients With Advanced NSCLC Diagnosed From October 1, 2016, Through December 31, 2018

Therapy	PD-L1 expression category, No. (% of total)			Patients tested with unknown expression status, No. (%)	Patients not tested, No. (%)
	0%	1%-49%	≥50%		
Any ICI	348 (32.3)	776 (40.3)	1541 (83.5)	310 (48.9)	755 (32.8)
ICI only	91 (8.4)	249 (12.9)	1264 (68.5)	160 (25.2)	409 (17.8)
Nivolumab	61 (5.7)	103 (5.4)	29 (1.6)	41 (6.5)	272 (11.8)
Pembrolizumab	16 (1.5)	124 (6.4)	1227 (66.5)	109 (17.2)	96 (4.2)
Atezolizumab or durvalumab^a^	14 (1.3)	22 (1.1)	10 (0.5)	10 (1.6)	41 (1.8)
ICI and chemotherapy	256 (23.8)	526 (27.3)	277 (15.0)	150 (23.7)	343 (14.9)
Chemotherapy alone	568 (52.7)	914 (47.5)	264 (14.3)	270 (42.6)	1351 (58.7)
Other	162 (15.0)	234 (12.2)	41 (2.2)	54 (8.5)	197 (8.6)
Total	1078	1924	1846	634	2303

Abbreviations: ICI, immune checkpoint inhibitor; NSCLC, non–small cell lung cancer; PD-L1, programmed cell death ligand 1.

^a^ Atezolizumab and durvalumab are combined to prevent cell sizes with fewer than 10 patients to prevent loss of confidentiality.

### Trends in ICI Use by PD-L1 Status

The proportion of patients receiving ICI treatment with low or untested PD-L1 expression increased over time. In the last quarter of 2016, 34 patients (13.2%) with negative or low expression received first-line ICI treatment, which increased to 709 (53.4%) in 2018. Among patients whose PD-L1 status was untested, use of first-line ICI treatment increased from 59 (17.0%) in the last quarter of 2016 to 394 (43.8%) in 2018. Use of ICIs remained high in all study periods among patients with PD-L1 expression of 50% or greater (109 [67.4%] in quarter 4 of 2016 and 655 [82.3%] in 2018). A total of 256 patients (73.6%) who received ICIs with negative or low PD-L1 expression did so in combination with chemotherapy, whereas 1264 (68.5%) of those with high PD-L1 expression were treated with ICIs as monotherapy. Among the subset of patients with negative PD-L1 expression who received an ICI in the first-line, 91 (26.1%) were treated with monotherapy.

We further examined use of first-line ICI treatment by PD-L1 expression status by NSCLC histologic type associated with the May 2017 accelerated approval for pembrolizumab in combination with chemotherapy for advanced nonsquamous NSCLC without restriction by PD-L1 status. Among patients with PD-L1 expression of 1% to 49%, use of first-line ICI increased from 23 (14.4%) in the first quarter of 2017 to 57 (37.5%) in the subsequent quarter. Although most ICI monotherapy was delivered to patients with PD-L1 expression of 50% or greater, there was persistent use among patients whose PD-L1 status was untested, negative (0%), or low (1%-49%) (eFigure 3 in the Supplement).

Among patients with advanced squamous NSCLC, off-label use of ICI therapy among patients with untested, negative (0%), or low (1%-49%) PD-L1 expression status persisted through the study period. Although the FDA restriction of first-line ICI treatment for patients with advanced squamous NSCLC was changed in the fourth quarter of 2018, there was increasing use of first-line ICI treatment that predated this broadened approval. In the quarter preceding the change in regulatory guidance, 33 patients (39.8%) with advanced squamous NSCLC without evidence of PD-L1 testing, 7 (29.2%) with negative (0%) PD-L1 staining, and 20 (36.4%) with PD-L1 expression of 1% to 49% received first-line ICI treatment.

## Discussion

We found that an increasing proportion of patients received first-line ICI treatment without evidence of PD-L1 testing and in the context of negative expression or levels below thresholds in place at the time. Despite evidence of ICI treatment that was discordant with regulatory PD-L1 recommendations in place at the time, most patients with high PD-L1 expression received ICI treatment consistent with the existing clinical indication. These findings suggest a high level of enthusiasm for the use of ICI treatment, often superseding recommendations for biomarker testing. We also observed that the proportion of patients receiving ICIs with negative or low PD-L1 expression increased during the study period, reflecting the accumulating evidence of clinical benefit. Taken together, these findings highlight the practical complexities associated with implementing a biomarker-based treatment strategy for new cancer drugs. These complexities may be particularly acute when test results are not clearly binary, and the science is rapidly changing because of the addition of new information that may affect interpretation of test results. Viewed from the context of an expanding role of molecular diagnostics, this work offers insights regarding the discordance between guidelines and real-world testing and treatment.^24^

Nearly one-third of patients without evidence of PD-L1 testing and an equal proportion with negative PD-L1 expression received ICI treatment in the first line. Within 3 months of approval, most newly diagnosed patients with advanced NSCLC received testing for PD-L1, suggesting that national practice was responsive to the recommendation to use PD-L1 status to select candidates for first-line ICI treatment. However, practice patterns increasingly favored ICI treatment in those without PD-L1 testing, lower levels of expression, or negative expression. Although pembrolizumab was granted accelerated approval for the first-line treatment of patients with advanced nonsquamous NSCLC in May 2017 without PD-L1 status restriction, we identified increasing use across all strata of PD-L1 expression in the period that preceded this accelerated approval.^10^ Moreover, first-line ICI therapy increased over time in the subset of patients with advanced squamous NSCLC before the expanded approval for this histologic type in October 2018. This finding may reflect an increasing inclination to treat with ICIs and tensions between clinical guidelines and their application to new immunotherapies.^25^

Most patients receiving first-line ICI treatment without PD-L1 testing or with expression levels beneath regulatory guidance thresholds also received other approved therapies. Although it may be encouraging that conventional treatment with known benefit was not omitted for most patients, the factors underlying discordant use of first-line ICIs are unknown. Prior evidence suggests that there is a high level of awareness of immunotherapy among patients with cancer and unrealistic expectations of these treatments buoyed by high-profile success stories.^26,27,28^ Furthermore, interest in these therapies may also be bolstered by direct-to-consumer advertising, although this effect on decision-making in advanced cancer treatment selection is unknown. Given potential costs and toxic effects, further research appears warranted to clarify the factors that underlie off-label use of expensive new cancer therapies. Additional efforts, including practitioner education and outreach, may be warranted to increase the use of complementary testing, if required, to select treatment.

### Limitations

The study sample was drawn from an enriched data set of patients treated in real-world settings. Although this approach affords contemporaneous assessment of care patterns that may be generalizable to the national population, there are several potential limitations.^20^ First, ascertainment of PD-L1 testing and test status was achieved by technology-enabled abstraction with multiple layers of quality assurance, including manual verification of PD-L1 reports with rigorous quality assurance processes. Nonetheless, there is a potential that testing occurring outside the Flatiron Health network was not captured, leading to underestimation of the prevalence of PD-L1 testing. However, we used several strategies to minimize this influence, including restricting the sample to patients actively engaged in care based on clinic visits and treatment, an approach previously applied.^29^ Because PD-L1 reports were also manually verified, we believe that there was only minimal potential for misclassification of test results reported as negative or low expression. Second, we used diagnosis codes from EHR data to assess comorbidity, an approach that may underestimate these measures associated with manual abstraction.^30^ Third, through the exclusive use of EHR data, we were not able to evaluate how patient or physician preferences altered these decisions. Therefore, we were unable to determine the contributions of new clinical evidence and patient preferences or physician judgement in the observed trends. Fourth, we did not evaluate clinical outcomes or costs associated with ICIs. Other limitations of all retrospective real-world data include the potential for missingness for critical variables associated with race/ethnicity and insurance status, misclassification of treatment exposure, and generalizability. Further investigation appears to be warranted given the potential for greater ICI treatment toxic effects in real-world settings compared with clinical trials.^31^

## Conclusions

During the period when regulatory guidance initially recommended that first-line ICI treatment be limited to patients with advanced NSCLC whose tumors express PD-L1, a substantial proportion of patients with negative, low, or untested PD-L1 expression status received first-line ICI treatment. The use of ICIs among patients with negative, low, or untested PD-L1 expression increased over time and paralleled the availability of new evidence that supported the efficacy of these agents across all strata of PD-L1 expression. These findings suggest that real-world practice was highly responsive to new clinical evidence and in a manner that preceded formal changes to regulatory guidance.

## Notes

Supplement. **eFigure 1.** Study Schema **eFigure 2.** Percent of Patients With Advanced Non-Small Cell Lung Cancer Receiving PD-L1 Testing by Quarter **eFigure 3.** Proportion of Patients Receiving First-Line Immune Checkpoint Inhibitor (ICI) Therapy Among 7,785 Patients With Advanced Non-Small Cell Lung Cancer (aNSCLC) by Subgroup: (A) Squamous Cell Histology, (B) Non-Squamous Histology, (C) ICI Delivered as Monotherapy Across All Tumor Histologic Subtypes, and (D) ICI Monotherapy Among Patients With Non-Squamous NSCLC **eTable 1.** Dates of Presentation and Publication of Pivotal Trials Addressing the Clinical Efficacy of First-Line Pembrolizumab **eTable 2.** Multivariable Logistic Regression Model Examining Factors Associated With PD-L1 Testing Among Patients in Flatiron aNSCLC Cohort (N = 7,785)
