# Supplementary material for: Association of Programmed Cell Death Ligand 1 Expression Status With Receipt of Immune Checkpoint Inhibitors in Patients With Advanced Non–Small Cell Lung Cancer
Source: JAMA Netw Open. 2020 Jun 8;3(6):e207205. doi: 10.1001/jamanetworkopen.2020.7205 (PMC7280954; doi:10.1001/jamanetworkopen.2020.7205)
Supplement: Supplement. — eFigure 1. Study Schema eFigure 2. Percent of Patients With Advanced Non-Small Cell Lung Cancer Receiving PD-L1 Testing by Quarter eFigure 3. Proportion of Patients Receiving First-Line Immune Checkpoint Inhibitor (ICI) Therapy Among 7,785 Patients With Advanced Non-Small Cell Lung Cancer (aNSCLC) by Subgroup: (A) Squamous Cell Histology, (B) Non-Squamous Histology, (C) ICI Delivered as Monotherapy Across All Tumor Histologic Subtypes, and (D) ICI Monotherapy Among Patients With Non-Squamous NSCLC eTable 1. Dates of Presentation and Publication of Pivotal Trials Addressing the Clinical Efficacy of First-Line Pembrolizumab eTable 2. Multivariable Logistic Regression Model Examining Factors Associated With PD-L1 Testing Among Patients in Flatiron aNSCLC Cohort (N = 7,785) [file jamanetwopen-3-e207205-s001.pdf]

Supplementary Online Content

Leapman MS, Presley CJ, Zhu W, et al. Association of programmed cell death ligand 1 expression status with receipt of immune checkpoint inhibitors in patients with advanced non–small cell lung cancer. *JAMA Netw Open*. 2020;3(6):e207205. doi:10.1001/jamanetworkopen.2020.7205

eFigure 1. Study Schema

eFigure 2. Percent of Patients With Advanced Non-Small Cell Lung Cancer Receiving PD-L1 Testing by Quarter

eFigure 3. Proportion of Patients Receiving First-Line Immune Checkpoint Inhibitor (ICI) Therapy Among 7,785 Patients With Advanced Non-Small Cell Lung Cancer (aNSLC) by Subgroup: (A) Squamous Cell Histology, (B) Non-Squamous Histology, (C) ICI Delivered as Monotherapy Across All Tumor Histologic Subtypes, and (D) ICI Monotherapy Among Patients With Non-Squamous NSCLC

eTable 1. Dates of Presentation and Publication of Pivotal Trials Addressing the Clinical Efficacy of First-Line Pembrolizumab

eTable 2. Multivariable Logistic Regression Model Examining Factors Associated With PD-L1 Testing Among Patients in Flatiron aNSCLC Cohort (N=7,785)

This supplementary material has been provided by the authors to give readers additional information about their work.

© 2020 Leapman MS et al. *JAMA Network Open*.

eFigure 1. Study Schema

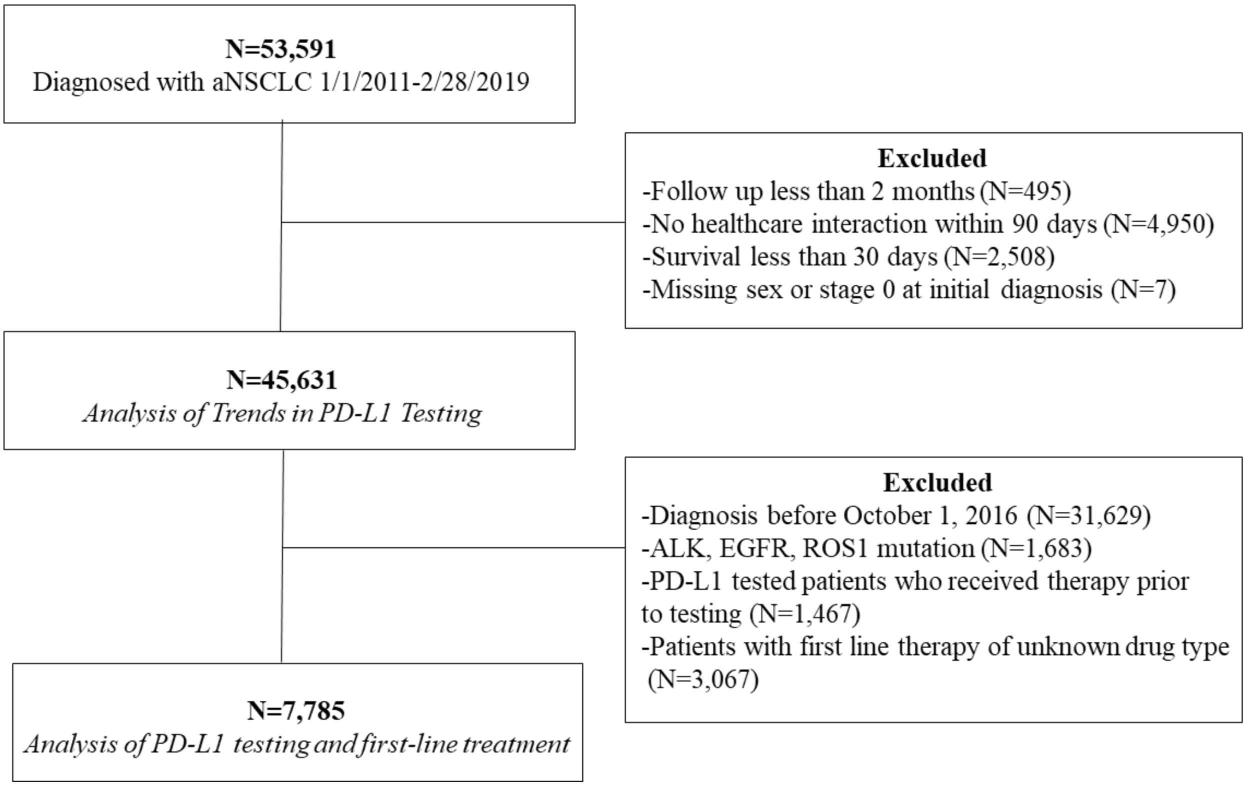

© 2020 Leapman MS et al. *JAMA Network Open*.

eFigure 2. Percent of Patients With Advanced Non-Small Cell Lung Cancer Receiving PD-L1 Testing by Quarter

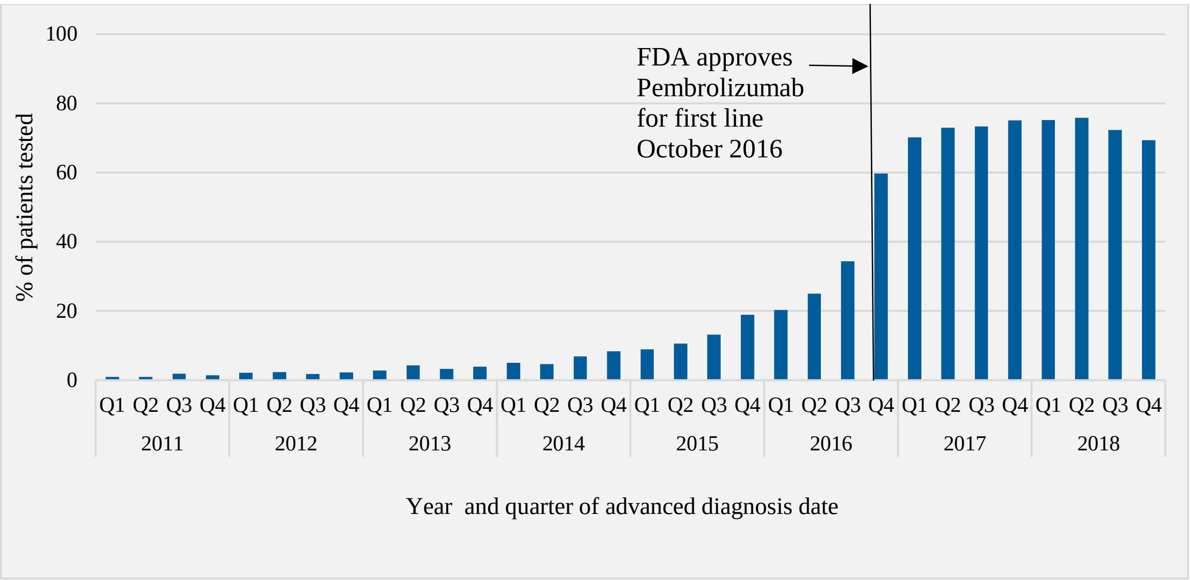

© 2020 Leapman MS et al. *JAMA Network Open*.

eFigure 3. Proportion of Patients Receiving First-Line Immune Checkpoint Inhibitor (ICI) Therapy Among 7,785 Patients With Advanced Non-Small Cell Lung Cancer (aNSLC) by Subgroup: (A) Squamous Cell Histology, (B) Non-Squamous Histology, (C) ICI Delivered as Monotherapy Across All Tumor Histologic Subtypes, and (D) ICI Monotherapy Among Patients With Non-Squamous NSCLC

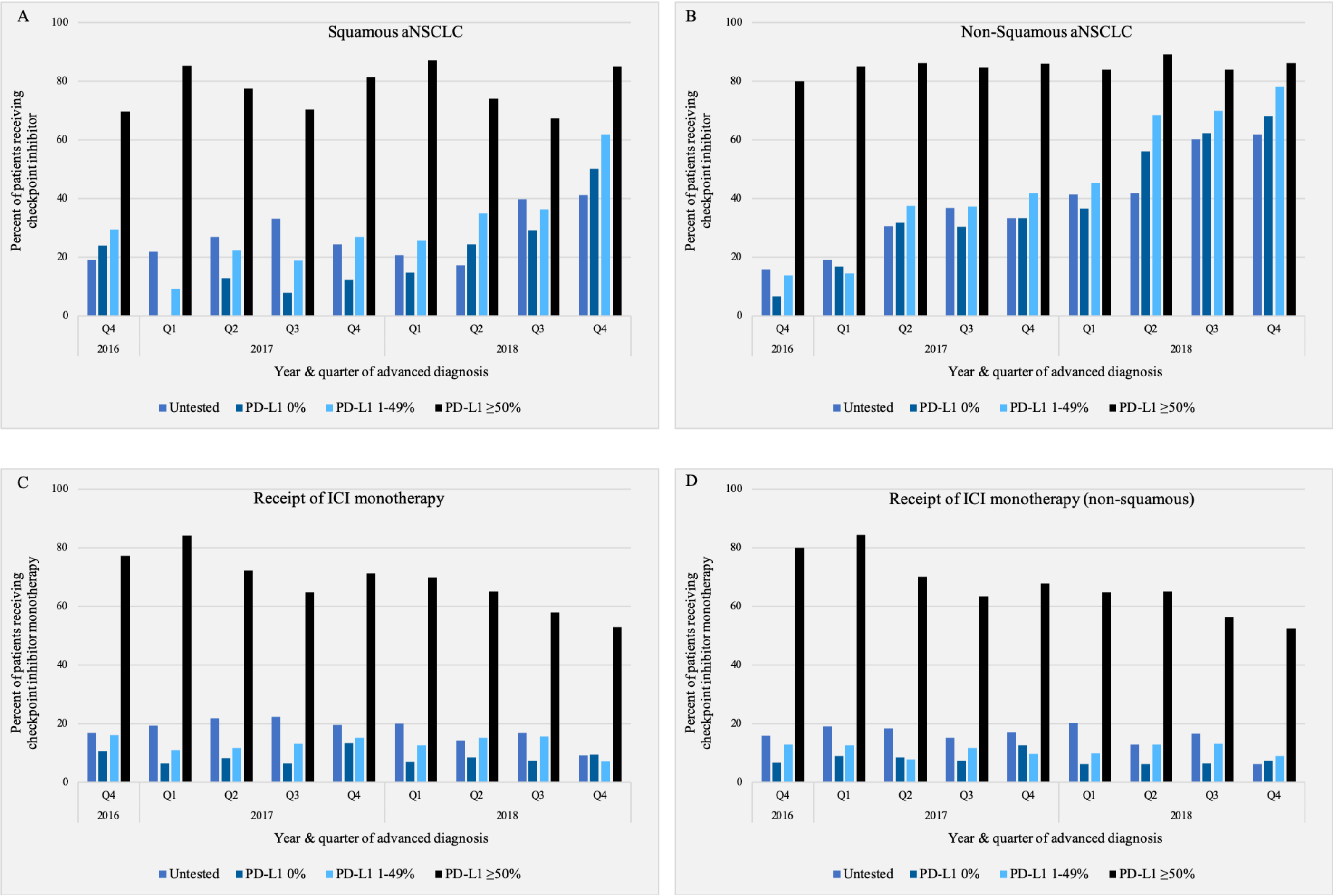

© 2020 Leapman MS et al. *JAMA Network Open*.

eTable 1. Dates of Presentation and Publication of Pivotal Trials Addressing the Clinical Efficacy of First-Line Pembrolizumab

Clinical Trial	Date of Presentation	Date of First Electronic Publication	Primary Findings	Associated FDA Regulatory Approval
KEYNOTE 024	Oct 9, 2016<br>ESMO Congress	October 24, 2016<br>New England Journal of Medicine [4]	Pembrolizumab associated with improved overall survival in patients with PD-L1≥50% versus chemotherapy	**October 24, 2016:** Pembrolizumab approved for treatment of patients with metastatic NSCLC whose tumors express PD-L1 (≥50%)
KEYNOTE 021(Cohort G)	June 2017<br>ASCO Annual Meeting	August 21, 2018<br>Journal of Thoracic Oncology [33]	Pembrolizumab plus pemetrexed and carboplatin improved objective response rates and progression-free survival in advanced non-squamous NSCLC regardless of PD-L1 expression	**May 10, 2017:** Accelerated approval for pembrolizumab in combination with pemetrexed and carboplatin for advanced non-squamous NSCLC
KEYNOTE 189	April 16, 2018*<br>AACR Annual Meeting	April 16, 2018*<br>New England Journal of Medicine [5]	Pembrolizumab in combination with pemetrexed and platinum-based chemotherapy prolonged overall and progression-free survival in patients with non-squamous NSCLC regardless of PD-L1 expression	**August 20, 2018:** Pembrolizumab approved in combination with pemetrexed and platinum-based chemotherapy for patients with advanced non-squamous NSCLC regardless of PD-L1 expression
IMpower 150	April 14-18, 2018<br>AACR Annual Meeting	June 14, 2018<br>New England Journal of Medicine [11]	The addition of atezolizumab to bevacizumab plus chemotherapy significantly improved progression-free survival and overall survival among patients with advanced non-squamous NSCLC, regardless of PD-L1 expression	**December 7, 2018:** Atezolizumab approved in combination with bevacizumab, paclitaxel and carboplatin for first-line treatment of advanced non-squamous NSCLC regardless of PD-L1 expression
KEYNOTE 407	June 3, 2018:<br>ASCO Annual Meeting	September 25, 2018<br>New England Journal of Medicine [6]	Pembrolizumab in combination with pemetrexed and platinum-based chemotherapy	**October 30, 2018:** Pembrolizumab approved in combination with carboplatin and either

© 2020 Leapman MS et al. *JAMA Network Open*.

			prolonged overall and progression-free survival in patients with squamous NSCLC regardless of PD-L1 expression	paclitaxel or nab-paclitaxel in patients with advanced squamous NSCLC regardless of PD-L1 expression
KEYNOTE 042	April 9, 2018: Press release after interim analysis by trial DMC<br>June 3, 2018: ASCO Annual Meeting	April 4, 2019 Lancet [7]	Among patients with advanced NSCLC whose tumors expressed PD-L1 (≥1%), pembrolizumab was associated with improved overall survival versus chemotherapy	April 11, 2019** Pembrolizumab approved for first-line treatment of patients with advanced NSCLC as monotherapy in patients with PD-L1 expression ≥%1

*Simultaneous presentation and publication; **FDA approval occurred outside time window of study; DMC=Data Monitoring Committee; ESMO=European Society of Medical Oncology; AACR=American Association of Cancer Research; PD-L1=programmed cell death ligand 1; NSCLC=non-small cell lung cancer

© 2020 Leapman MS et al. *JAMA Network Open*.

eTable 2. Multivariable Logistic Regression Model Examining Factors Associated With PD-L1 Testing Among Patients in Flatiron aNSCLC Cohort (N=7,785)

	Odds Ratio	95% Confidence Interval		P Value
		Lower	Upper	
**Age**				
≤45	ref	--	--	--
46-55	0.95	0.55	1.64	0.86
56-65	0.95	0.57	1.60	0.86
66-75	1.20	0.69	2.09	0.53
76-85	1.24	0.71	2.18	0.45
**Sex**				
Male	ref	--	--	--
Female	1.22	1.10	1.36	<.001
**Race**				
Non-Hispanic White	ref	--	--	--
Non-Hispanic Black	0.87	0.73	1.04	0.14
Hispanic/Latino	0.84	0.63	1.11	0.21
Asian	1.12	0.73	1.70	0.61
Other	0.96	0.80	1.17	0.71
Missing	0.73	0.62	0.86	<.001
**Insurance**				
Medicare and other	ref	--	--	--
Medicare only	1.11	0.91	1.37	0.30
Medicare, unknown	1.03	0.88	1.20	0.76
Commercial Health Plan	1.21	0.94	1.56	0.14
Medicaid	1.06	0.61	1.85	0.84
Other	1.19	0.88	1.60	0.26
Uninsured/unknown	1.03	0.79	1.34	0.84
**Histology**				
NSCLC histology NOS	ref	--	--	--
Non-squamous cell carcinoma	1.68	1.34	2.10	<0.001
Squamous cell carcinoma	1.05	0.83	1.33	0.69
**Stage at Initial Diagnosis**				
Stage I	ref	--	--	--
Stage II	0.95	0.73	1.25	0.73
Stage III	0.68	0.56	0.83	<0.001
Stage IV	1.88	1.56	2.26	<0.001
Not reported / Occult	1.04	0.81	1.33	0.79

© 2020 Leapman MS et al. *JAMA Network Open*.

**ECOG score**				
0	ref	--	--	--
1	1.19	1.04	1.35	0.009
2	1.14	0.96	1.36	0.14
3-4	1.34	0.97	1.86	0.07
Missing	1.11	0.96	1.29	0.15
**Number of comorbid conditions**				
0	ref	--	--	--
1-2	1.07	0.91	1.26	0.42
3+	1.15	0.86	1.52	0.35
**Smoking Status**				
Yes	ref	--	--	--
No	0.94	0.77	1.15	0.56
Unknown	0.13	0.03	0.50	0.003
**Diagnosis Year**				
2016	ref	--	--	--
2017	1.93	1.64	2.27	<0.001
2018	2.14	1.81		<0.001

© 2020 Leapman MS et al. *JAMA Network Open*.
